# Commercial Translation of Electrochemical Biosensors: Supply Chain Strategy, Scale-Up Manufacturing, and Regulatory–Quality Considerations

**DOI:** 10.3390/bios16020112

**Published:** 2026-02-09

**Authors:** Gao Zhou, Haibin Liu

**Affiliations:** 1 School of Management, Hunan University of Information Technology, Changsha 410151, China; gaozhou86@gmail.com; 2 Institute Montpellier Management, University of Montpellier, 34960 Montpellier, France; 3 Graduate School of International Studies, Yonsei University, Seoul 03722, Republic of Korea

**Keywords:** scale-up manufacturing, regulatory strategy, quality management systems, material sourcing, clinical translation

## Abstract

Electrochemical biosensors have reached a high degree of analytical maturity; however, only a small portion of laboratory demonstrations actually progress to commercial products. In this review, we looked non-analytically at the factors which are in place with respect to this translational gap, specifically looking into supply chain design, scale-up manufacturing strategy, regulatory–quality, and more. Based on a wide range of academic and industrial literature, the paper considers how decisions about what kind of material to use, especially for material that recognizes living things, conductive material made from ink, and the material that is the actual product being made, can make a big difference in whether the product can be reproduced easily, if it will stay stable for a long time, and if it is allowed according to the rules. This review compares the dominant manufacturing paradigms—roll-to-roll printing, and semiconductor-derived microfabrication—and shows how the respective strengths and limitations match the different targets, costs, and risk class. This is more about making manufacturing an upstream optimization problem than treating processes as defects and quality as assurance, rather than making it an upstream optimization problem. And it does this by looking at some other big pathways for regulations in the U.S., EU, and China as well, where we get to see how those differences in classification requirements, what kind of proofs you should have, and different ways about running those quality management systems affect how quickly things can come out after developing them, and what your flexibility with customers is like when those products are already out there in the world. The study looks at some case studies: disposable glucose strips, cartridge-based blood analyzers, and new continuous monitoring systems are used to show how the exact same electrochemical ideas can result in very different commercialization issues based on the clinical context and system integration. Synthesizing those angles creates a review that can give a system level map of matching research design to industrial and regulatory realities, with the goal of making it easier for electrochemical biosensors to go from lab prototypes to ready-for-market diagnostic tools.

## 1. Introduction

Electrochemical biosensors are one of the most promising and established classes of biosensing technology, based on decades of intensive research showing promise for applications in clinical diagnostics, environmental monitoring, food safety, and defense [1,2]. They have inherent advantages like being highly sensitive and selective, having fast response times, being cheap, and being able to be made smaller, so they are good for POC testing and wearable continuous monitoring devices [3,4]. As the archetypal success story of the screen-printed glucose biosensor, which changed the face of diabetes treatment and made a multi-billion-dollar industry, it serves as evidence of the commercial viability of these sensors [5,6]. This translational discontinuity is often described as the “valley of death,” which, in the biosensor context, refers to the high-risk gap between a laboratory prototype and a clinically usable, scalable, and compliant device—one that can be manufactured reproducibly, supplied reliably, validated in real sample matrices and workflows, and supported by a regulatory-grade quality system over the full product life cycle [7]. Scientific novelty alone frequently fails to bridge this gap because the main barriers are system-level and evidence-driven: the analytical performance demonstrated in buffers does not guarantee lot-to-lot consistency after scale-up, stability across distribution and shelf life, robustness to interferents and user handling, or traceability under design-control and risk-management requirements. In addition, the transition requires substantial resources for process development, verification/validation, clinical performance evidence, and regulatory submissions, where financing and execution capacity commonly become limiting even when the underlying sensing chemistry is strong [8]. Consistent with the scope of this review, these constraints make supply chain strategy, scale-up manufacturing, and regulatory–quality planning central determinants of whether electrochemical biosensors cross the valley of death rather than optional downstream steps. This large gap between academic innovation and market realization, often referred to as the valley of death, is not just because of scientific or technical issues, but also involves difficulties in manufacturing, logistics, and regulations [9].

**Figure 1:**
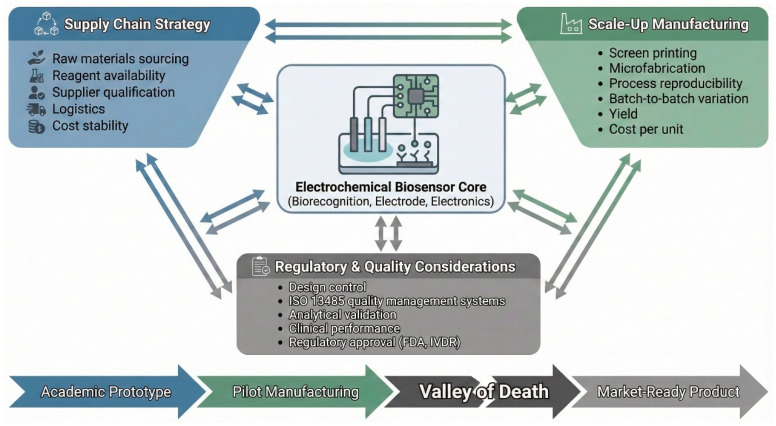
System-level overview of the commercial translation of electrochemical biosensors.

The successful commercial translation of an electrochemical biosensor is far more than the sum of the parts, which are the biorecognition and the transduction elements. As shown in Figure 1, the commercial translation of electrochemical biosensors occurs over a finely coupled system including supply chain strategy, scalable manufacturing, and regulatory–quality that must be tackled in parallel at the outset of the technological development. It demands an all-encompassing and forward-thinking approach which includes supply chain resilience, scalable and reproducible manufacturing, and negotiating stringent global regulatory landscapes at the very start of the development process [10]. It is not surprising that so many good technologies are unable to get past the practical and strategic hurdles: research labs [11]. For example, a novel biosensor might have femtomolar detection limits in a buffered solution, but whether or not that makes sense commercially depends on if you can mass manufacture with a less than 5% batch-to-batch variation, if your key reagents can be sourced reliably and cheaply, and if it will meet the tough performance and safety standards set by places like the U.S. FDA or EU’s IVDR [12].

This review paper will offer a critique and full consideration over the vital non-scientific issues and tactics for the commercial transformation of electrochemical biosensors. We shift our attention away from the basic ideas of biosensor building, and we now look at three important parts of selling this product: first, we are looking at the supply-chain aspect, so that means getting the parts, buying them, and making sure the delivery system is strong enough. Second, we are thinking big here, so if we make lots of products quickly or we carefully make them one by one, it actually changes how well they come out and how much money they cost. Third, we get to the big part that cares about following rules and doing a quality job so people can buy it, and we make some sense about the big rules and tools used by everyone. By seeing what the scholars say, what the problem actually is, and what needs to be done in each of those areas, I am going to show people how to do research and start up businesses and work in companies if they want, so they can help it be able to make correct diagnoses and do its work in the real world, not just in a lab. This article is a structured narrative review with scoping elements designed to map translational risks across the commercial lifecycle of electrochemical biosensors. Literature sources were identified through targeted searches conducted in Scopus, Web of Science, PubMed, and IEEE Xplore, complemented by the forward/backward citation screening of highly cited translation and manufacturing papers and by searches of regulator and standards-body websites for primary guidance documents. The search strings combined terms for electrochemical biosensors with commercial translation concepts (e.g., ‘electrochemical biosensor’ AND ‘scale-up’ OR ‘manufacturing’ OR ‘roll-to-roll’ OR ‘quality management system’ OR ‘ISO 13485’ OR ‘FDA 510(k)’ OR ‘IVDR’). The inclusion criteria were English-language sources that addressed at least one of the three focal domains (supply chain, manufacturing scale-up, and regulatory–quality) with explicit relevance to biosensors or in vitro diagnostics; the exclusions were purely analytical-performance papers with no manufacturing or regulatory content. Records were screened at the title/abstract level, and then the full-text level to confirm relevance, with disagreements resolved by author consensus. The reporting of the review workflow is informed by the scoping review reporting guidance (PRISMA-ScR) to improve transparency even though the intent is mapping and synthesis rather than meta-analysis.

## 2. Supply Chain Strategy for Electrochemical Biosensor Production

Robust and efficient supply chains are necessary for any medical device to be commercially successful, but it is often one of the first things to be ignored in the development of electrochemical biosensors [13]. A good supply chain strategy makes sure that the material is available in a timely and cost-effective manner, as well as ensures product quality, regulatory compliance, and resilience to geopolitical or economic disruption [14]. For electrochemical biosensors which usually require a certain combination of biological reagents, special chemicals, and electronic components, the management of the supply chain faces special difficulties that need to be handled proactively [4].

Sourcing and purchasing raw materials are an important component of the supply chain strategy. Electrochemical biosensors are composite devices made up of a variety of materials ranging from simple plastic housing to more specific, and often delicate, biological molecules such as enzymes and antibodies and conductive inks and functionalized nanomaterials [15]. As all of the research paper show, the quality and purity of the input materials to the device have a very pretty affect on the final performance of the device [15]. The debate over single sourcing vs. multi-sourcing is most pertinent. A central strategic choice is whether to rely on single-sourcing or to implement dual-/multi-sourcing for materials such as biorecognition elements, conductive inks, polymer substrates, and electronic components. Single-sourcing can be rational early in translation because it reduces the supplier-qualification workload, supports a tighter material/process capability, and often yields a lower unit cost through volume commitments and simplified incoming inspection. However, its failure mode is systemic: when a disruption affects the sole source or its upstream tiers, the biosensor manufacturer faces immediate line stoppage, forced component redesign, or emergency revalidation—each of which can trigger significant quality/regulatory risk in medical devices. The COVID-19 pandemic highlighted that these disruptions are frequently correlated and geographically clustered rather than independent. Lockdowns and workforce constraints reduced capacity in specific regions; international transport bottlenecks and reduced air freight capacity delayed time- and temperature-sensitive shipments; and export controls or ‘domestic prioritization’ policies constrained access to medical supplies and components even when nominal production capacity existed. These coupled shocks produced well-documented scarcities in healthcare supply chains and exposed how the over-reliance on a concentrated set of countries or regions can propagate shortages downstream in medical products, including diagnostics and devices. In contrast, multi-sourcing that is explicitly geo-diverse functions as a resilience investment: it lowers the probability that a single regional shock simultaneously disables all supply options and allows for a faster substitution when one node fails. Empirical evidence during the COVID-19 crisis also indicates that the diversification of supply bases improved firms’ ability to maintain supply and performance under disruption and recovery conditions. For electrochemical biosensors, this strategy is especially important for (i) biologics and biochemicals with cold-chain constraints and lot-to-lot variability that can shift calibration or sensitivity, and (ii) electronics subject to long lead times and allocation during global shortages, making a geo-diverse supplier base a practical requirement for the continuity of manufacturing and regulatory-compliant change control rather than an optional cost add-on [16]. This is especially important for bioreagents, where lot-to-lot variability can originate from shifts in critical molecular attributes. These changes directly propagate into electrochemical performance by shifting the effective recognition/catalysis kinetics, which, in turn, alters the calibration slope/intercept, LOD/LOQ, and precision across production lots. Immunoassay experience shows that antibody lot-to-lot variance can measurably change the assay background and apparent analyte concentration, creating clinically relevant between-lot bias if not controlled [17]. More broadly, laboratory medicine has identified reagent/calibrator lot changes as a frequent cause of result shifts with potential clinical consequences, reinforcing the need for formal lot verification and acceptance criteria rather than informal “equivalency-by-supplier” assumptions [18]. Therefore, the incoming quality control for biosensor bioreagents must be performance-linked: beyond documentation and shipment checks, each lot should be released using predefined functional activity specifications plus electrochemical benchmarking against internal reference materials/controls to detect lot-induced drift before scale manufacturing.

Robust and efficient supply chains are necessary for any medical device to be commercially successful; yet, supply-chain constraints also matter scientifically because they determine whether an assay can be reproduced across laboratories, whether performance improves lots, and whether early prototypes can be credibly scaled into regulated products. In electrochemical biosensors, upstream sourcing decisions are tightly coupled to downstream analytical variability because key inputs directly affect the calibration slope, limit of detection, inter-batch reproducibility, and shelf life. For medical products, diversification measures such as parallel sourcing are widely recognized as core resilience mechanisms, but diversification is only effective when sources are independent, because correlated failures can negate redundancy benefits. In this context, the single-sourcing versus multi-sourcing decision should be treated as an explicit cost–risk tradeoff rather than a purely ‘industrial procurement’ choice. Purchasing research shows that single sourcing can amplify the exposure to supplier default or disruption under uncertainty, whereas multiple sourcing reduces that exposure at the cost of higher supplier qualification, coordination, and quality-management overhead [19]. For biosensors with translational intent, we therefore recommend a risk-based minimum of dual sourcing (≥2 qualified and independent suppliers) for critical biorecognition elements and other ‘single-point-of-failure’ inputs whenever feasible, together with centralized IQC and electrochemical benchmarking to detect lot-induced shifts before scale manufacturing. This recommendation is not limited to large manufacturers. Researchers can operationalize it early by selecting reagents with at least one credible second source, documenting functional specifications, and performing lot-to-lot robustness experiments that quantify the sensitivity drift across lots and/or suppliers. When dual sourcing is not feasible, researchers should preserve optionality by validating a second synthesis route or alternative receptor format and by defining the acceptance criteria that later enable supplier qualification without redesigning the assay. Evidence from healthcare supply disruptions during COVID-19 further highlights that availability is improved not only by buffering but also by bridging strategies such as long-term supplier relationships and procurement support mechanisms, reinforcing the need to treat supplier quality and collaboration as part of the technical development plan rather than a late-stage purchasing activity [13].

**Figure 2:**
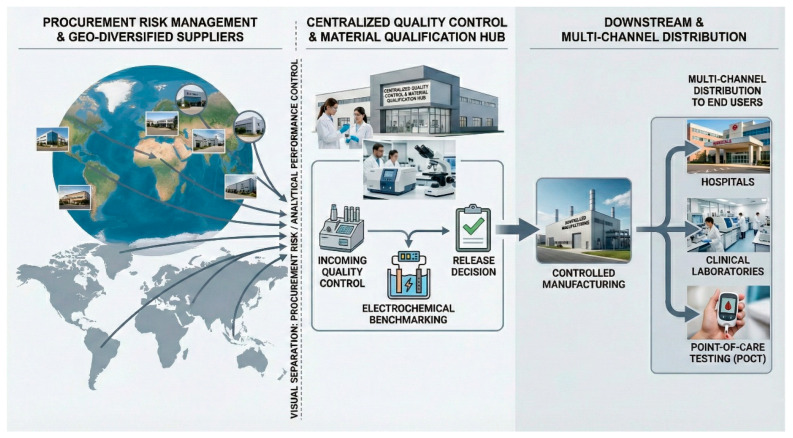
Conceptual diagram of a resilient supply chain for electrochemical biosensor manufacturing, illustrating geo-diversified sourcing for critical components like antibodies and enzymes, centralized quality control, and multi-channel distribution to end-users.

**Figure 3:**
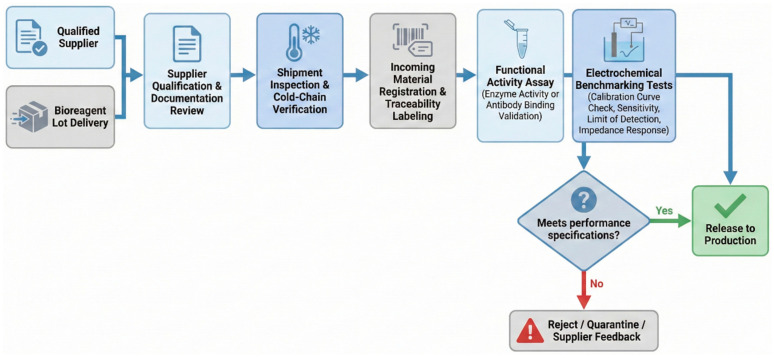
A flowchart depicting the IQC process for a critical bioreagent, showing steps from supplier qualification and shipment verification to functional testing and release to production.

A resilient biosensor supply chain is schematically illustrated in Figure 2. This framework deliberately decouples the procurement risk from the analytical performance risk by combining the geographically diversified sourcing of critical materials with centralized quality control and release decision-making. Enzyme suppliers, antibody suppliers, and electronic component manufacturers are distributed across multiple regions to minimize the impact of localized disruptions. In contrast, material qualification, calibration, and electrochemical performance benchmarking are centralized to ensure consistency, comparability, and regulatory traceability. To further systematize this concept, Table 1 summarizes the principal materials involved in electrochemical biosensor fabrication and maps dominant supply chain risks to the corresponding mitigation strategies. Importantly, supply chain considerations extend beyond logistics and availability; they directly influence analytical robustness and device performance. For example, variability in biorecognition elements can propagate into shifts in calibration sensitivity, limits of detection, and inter-batch reproducibility, necessitating rigorous incoming activity assays and standardized electrochemical benchmarking to identify and control such effects. This performance-linked quality control is operationalized by a structured IQC workflow for critical bioreagents, as summarized in Figure 3. Upon receipt, each lot is first placed in quarantine and subjected to identity and shipment-condition verification, including the confirmation of the chain of custody, cold-chain indicators, labeling, expiry, and certificate-of-analysis consistency with the supplier quality agreement. A statistically defined sampling plan is then applied to generate retain samples and test aliquots that support both immediate lot disposition and long-term traceability. The lot is next evaluated for biochemical fitness-for-use using orthogonal functional assays appropriate to the reagent class, such as enzyme activity, binding performance metrics for affinity reagents, and critical impurity screens when relevant, recognizing that reagent lot-to-lot variability is a documented driver of irreproducibility and analytical drift in antibody- and immunoassay-based measurements [20].

Crucially, biochemical assays alone do not fully predict device-level behavior because electrochemical sensors convert biorecognition into an electrical signature that is sensitive to immobilization yield, interfacial charge transfer, and background electrochemistry. Therefore, we define electrochemical benchmarking as a standardized, lot-release test battery performed on a controlled ‘reference’ sensor build using fixed assembly steps, defined buffers, and a calibrated potentiostat protocol, so that new lots can be quantitatively compared against a qualified ‘golden’ lot and historical control limits. Benchmarking typically includes (i) electrochemical baseline checks, (ii) interface-sensitive metrics such as impedance-derived charge-transfer resistance and capacitance trends (EIS) for detecting altered interfacial kinetics or fouling propensity, and (iii) analyte-relevant calibration checks, with the acceptance criteria pre-specified as part of the design history file and quality management system. This device-relevant benchmarking step is motivated by prior metrological analyses of printed electrochemical biosensors and empirical evidence that electrochemical performance can vary meaningfully even across commercially available electrode platforms, reinforcing the need for electrochemical performance qualification rather than paperwork-only receipt inspection [21]. Lot disposition is then determined by combining biochemical functionality results with the electrochemical benchmark performance against predefined limits; the results are trended across lots to enable the early detection of gradual drift, and retain samples are stored to support investigations, CAPA, and change-control activities. Where applicable, the lot-change evaluation logic is aligned with established principles used in regulated measurement settings for detecting clinically significant lot differences, while being implemented upstream at the manufacturer level to prevent performance shifts from entering scale-up manufacturing. Table 1 makes it clear by explicitly mapping each material class to both supply chain vulnerability and performable consequences, so that the supply chain strategy must be co-designed with materials characterization and quality control rather than viewed as a procurement function executed downstream.

**Table 1:** Key material categories in electrochemical biosensor translation, associated supply-chain risks, and TRL-specific mitigation strategies and best practices.

Material Category	Representative Examples	Primary Supply-Chain Risks	TRL Stage	Mitigation Strategies and Best Practices	References
Biorecognition Elements	Enzymes (GOx, LOx), antibodies, aptamers, peptides	Batch-to-batch activity variability; cold-chain disruption; limited GMP suppliers; shelf-life uncertainty	TRL 1–4	At early research and proof-of-concept stages, biorecognition elements should be screened from multiple suppliers and lots to establish baseline activity ranges and degradation pathways. Activity normalization (e.g., units per mg protein) and stress testing under temperature and humidity excursions help identify sensitivity to transport and storage conditions before scale-up.	[22,23]
			TRL 5–7	During pilot manufacturing, lot-to-lot qualification protocols should be introduced, including acceptance criteria based on electrochemical response (sensitivity, baseline current, and signal drift). Supplier change notification and retain-sample programs become critical to avoid untracked performance shifts.	
			TRL 8–9	At commercial scale, sourcing should be restricted to GMP-capable or ISO-certified suppliers with formal quality agreements. Bridging studies are required for any lot or supplier change, and long-term stability data must support labeled shelf life under defined storage conditions.	
Electrode Materials and Functional Inks	Carbon, graphene, CNT inks; Au/Pt inks; conductive polymers	Noble-metal price volatility; nanomaterial aggregation; formulation drift; printing reproducibility	TRL 1–4	Critical-to-quality attributes such as solids content, viscosity, particle size distribution, and post-cure sheet resistance should be defined early. Incoming lots should be benchmarked against a reference electrode using standardized redox probes to detect electrochemical variability arising from formulation differences.	[24,25,26]
			TRL 5–7	Supplier quality agreements should explicitly cover formulation changes, precursor substitutions, and dispersion protocols. Second-source qualification and standardized ink preparation (mixing energy, dispersion time, and aging window) reduce dependence on single vendors and minimize batch-induced variability.	
			TRL 8–9	At commercialization, statistical incoming inspection and retain-sample programs are recommended. For noble-metal inks, price-risk planning and dual sourcing mitigate cost shocks that could otherwise force unvalidated redesigns.	
Substrates and Structural Materials	PET, PEN, PI, paper, elastomers	Surface-energy variability; dimensional instability; humidity sensitivity; supplier consolidation	TRL 1–4	Substrates should be treated as functional components rather than passive supports. Early testing should define acceptable ranges for surface energy, roughness, thickness, and dimensional tolerance that directly affect printing yield and electrode adhesion.	[27]
			TRL 5–7	Multiple medical-grade or electronics-grade suppliers should be qualified, with controlled surface treatment (e.g., corona or plasma) locked to specific process parameters. Incoming substrate lots should be verified using rapid QC tests such as contact angle or dyne level.	
			TRL 8–9	Change-control procedures must cover resin formulation, additive packages, and finishing steps. For cellulose-based substrates, moisture conditioning and barrier packaging become essential to maintain shelf life and electrochemical consistency.	
Reagents, Mediators, and Chemicals	Buffers, redox mediators, crosslinkers, preservatives	Purity variability; oxidation or degradation; regulatory restrictions; hazardous handling	TRL 1–4	Fit-for-use grades and impurity tolerances should be established, especially for redox mediators and chemicals influencing background current. Lot changes should be screened electrochemically to detect subtle shifts in baseline or sensitivity.	[28,29,30]
			TRL 5–7	Formal lot-to-lot verification and acceptance criteria should be implemented, reflecting practices used in regulated analytical laboratories where reagent lot changes are recognized sources of measurement bias. Supplier change notification and retain-sample programs support traceability and root-cause analysis.	
			TRL 8–9	Long-term contracts with qualified suppliers, controlled storage conditions, and full documentation of hazardous reagent handling are required to prevent supply interruptions or compliance-driven production halts.	
Electronic Components and Modules	ASICs, BLE chips, batteries, connectors	End-of-life (EOL) risk; counterfeit parts; silent BOM changes; geopolitical supply shocks	TRL 1–4	Early designs should avoid single-source components by using pin-compatible alternatives and derating strategies. Component criticality should be documented to prioritize risk mitigation for safety- or performance-relevant parts.	[28,31]
			TRL 5–7	Authorized-channel procurement and counterfeit-avoidance practices (traceability, and incoming inspection where risk is-justified) should be adopted. EOL monitoring and last-time-buy planning reduce forced redesign during pilot deployment.	
			TRL 8–9	At commercial scale, supplier quality agreements must include hardware and firmware revision control with mandatory change notification. Component substitutions should trigger formal impact assessments and, where required, partial revalidation to ensure field reliability.	

Moreover, material selection has direct implications for supply-chain resilience and commercial execution, not only for environmental impact. A growing body of work positions paper and cellulose-derivative substrates as practical platforms for electrochemical sensing because they are low-cost, widely available, and compatible with scalable printing workflows, which collectively expands the feasible supplier ecosystem for substrate converting, printing, and packaging operations [32]. From a supply-chain perspective, the strategic value is that cellulose-based substrates draw from a geographically distributed, high-volume global pulp-and-paper industry, which improves second-sourcing options, reduces single-region dependency, and enables regionalized procurement that shortens lead times and lowers disruption exposure. This differs from specialty polymers or noble-metal intensive designs that can concentrate purchasing in a narrower vendor base and amplify price/availability shocks.

Transitioning to ‘green’ materials can also reduce the compliance burden and accelerate market access. In the EU, RoHS substance restrictions apply to medical devices and IVDs, creating ongoing requirements for restricted-substance control through supplier declarations, verification testing, and change-control governance; simplifying the material bill-of-materials toward bio-based substrates and well-characterized carbon-based conductors can reduce documentation complexity and the risk that a supplier material change triggers requalification. In parallel, sustainability is increasingly embedded in procurement practice, meaning that material choices can influence tender competitiveness and adoption in institutional customers that evaluate the lifecycle and hazardous-substance attributes as the award criteria.

Importantly, these supply-chain advantages do not imply that paper/cellulose is universally superior; rather, they shift the optimization landscape. Paper/cellulose platforms can trade extreme microfabrication resolution for manufacturability, supplier diversity, and simplified end-of-life pathways. Consequently, ‘green’ materials should be framed as a translation strategy that simultaneously manages procurement risk, process scalability, and compliance friction, instead of being treated as a downstream sustainability add-on [33].

Inventory management is also very important. Though JIT manufacturing seems promising in reducing warehousing costs, it is extremely risky for a product such as a medical device where a product not being available at the right time can literally be a matter of life and death [34]. A hybrid “Just in Case” strategy is to build up stockpiles of critical, long-lead, volatile-supply items for a buffer against the unforeseen disruption, but with no unnecessary carriage [35]. For the makers of electrochemical biosensors, that would translate into keeping a six-month supply on hand of a custom-synthesized redox mediator, say, or a given monoclonal antibody, while JIT-ing standard resistors, substrates, etc. At the end of the day, it is a proactive approach balancing costs, quality, and risk for a strong supply chain to have dependable diagnostic devices consistently.

## 3. Scale-Up Manufacturing: From Lab Bench to Mass Production

Scaling from a laboratory demonstration to regulated production at 10^5^–10^8^ units is often the main engineering barrier in commercial translation because it couples throughput, unit cost, and process capability into a single constraint space [10,11]. In practice, electrode manufacturing is not limited to a binary choice between printing and microfabrication. Instead, the landscape is better represented by three families of processes that differ in their manufacturing intent: high-throughput web-based printing, and high-precision wafer-based microfabrication that are increasingly used to prototype—and, in some cases, to manufacture—electrochemical devices with non-planar architectures. Recent perspectives explicitly position screen-printing and additive manufacturing as complementary rather than competing toolkits, with the key differentiators being achievable resolution, throughput, and the maturity of in-line quality control for regulated production [36].

**Figure 4:**
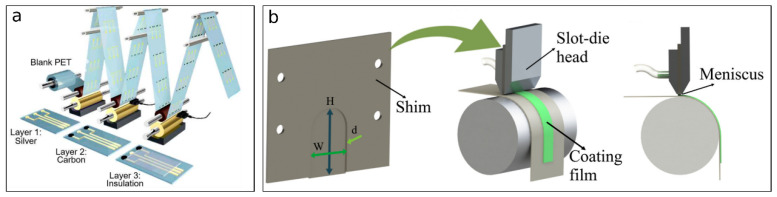
(**a**) Roll-to-roll gravure printing of biocompatible electrode arrays on flexible PET substrate allows high-throughput, low-cost production of sensing electrodes that can be fabricated with controllable size and density [37]. (**b**) Roll-to-roll processing technique: shim with dimensions H (height), w (width), and d (depth); slot-die head and coating film; and meniscus between slot-die head and substrate.

Roll-to-roll printing remains the dominant economic pathway for disposable strips because it supports continuous, high-yield manufacturing at very low unit cost; for example, commercial glucose test strips have been reported as high-volume products (billions per year) manufactured at low cost (5–15 cents per strip) with very low defect rates (<0.1%) in mature production environments [37,38,39]. At the same time, additive manufacturing methods (e.g., fused-filament fabrication, stereolithography, and direct-ink writing) are rapidly expanding electrode design freedom (3D features, rapid iteration, and embedded fluidics), but their scale-up constraints differ: throughput is typically lower than R2R, and material qualification plus post-processing control often dominate variability and regulatory risk [40]. Other than the standard glucose-strip sort of narrative, there is also a translationally relevant example of an R2R capability, provided by Bariya et al. [37], who reported R2R gravure-printed electrochemical electrodes on 150 m flexible substrate rolls and demonstrated that such printed electrodes (Figure 4a) can be functionalized into consistent performing sensors for multiple targets (ions, metabolites, heavy metals, and small molecules) in wearables/medical settings, positioning uniform electrochemical kinetics across long rolls as a requisite for scale manufacture rather than a laboratory oddity. The primary argument for R2R is economic: it has high-throughput fabrication, reduces material waste, and can be made into flexible/disp. devices [41,42]. But those who oppose and doubt have many worries about the control process and uniformity [43]. To obtain a uniform layer thickness and exact layer-to-layer registration as well as the consistent electrochemical activity of deposited inks over hundreds of meters of a moving web is technically challenging [41,44]. It is important to note that the manufacturing risk is not limited to the conductor patterning step: in many biosensors, the biofunctional layer becomes the largest source of variability between lots as it is deposited and dried, a history that couples directly to the amount of activity retained and how much the signal disperses. Here, Cagnani et al. [45] demonstrated what I shall be calling a “hybrid R2R,” where we start with our screen-printed electrodes and then do a subsequent R2R slot-die coating, which includes material like our enzyme-containing inks (Figure 4b), which had a very good reproducibility, and explicitly argue that slot-die deposition can be used to print enzymes without a large activity loss, which is a reasonable process path to reduce biological variability in a large run. But there are little challenges like changes in the ink’s viscosity, the nozzle getting blocked up, or the screen getting all messed up that sneak in and cause a batch-to-batch performance swing, which is a major point of failure for getting the go-ahead from regulators [46,47]. While these techs are good at getting easier layered electrode geoms, it is hard to make those sharp high-res 3D shapes that can be made in other ways [39]. To make this tradeoff clearer, Table 2 makes a comparison of the R2R printing and microfabrication methods on many levels: work, prices, resolution, and consistency. The table does not present a simple binary judgement. Instead, it shows that the real tension between these approaches is not about technological maturity, but rather about manufacturing intent. R2R rocks if the target product’s use case profile calls for ULTRA high volume, a mechanical flexible product, and a really aggressive cost tradeoff for having features at a high reset. Contrariwise, microfabrication provides better geometric fidelity and interdevice uniformity so that, when electrochemical performances have sensitivities toward the diffusion length scales, electric fields’ confinement levels, or uniformities of electrode–electrolyte interfaces, it turns out to be critical. And, importantly, Table 2 shows that batch-to-batch reproducibility and regulatory robustness tend to be underweighted in the early-stage academic demos and are the most crucial aspects during commercial translation. The table shows that, although printing approaches are often referred to as “simpler”, the process is more challenging to control over a longer production run. On the contrary, although the microfabrication has a higher upfront capital expenditure, it can lower the downstream quality risk because its tight control over the unit operations. And this kind of comparison serves as a kind of prop-up for the main argument which is that the manufacturing strategy should be picked backwards from what you want to happen clinically, with the regulators, and economically with the final device, not from what is most convenient in the lab.

On the other hand, the other side of the debate includes techniques of microfabrication [48]. Microfabrication’s big selling point is being exact. These methods can be used to create electrode features at a less than micron resolution. They make it possible to produce highly reproducible, small, and complicated arrays of sensors, which is impossible to do with normal printing [49]. A practical instance of a “precision dividend” is the creation of interdigitated microelectrodes featuring micron-scale gaps and geometry that has been controlled: Støvring et al. [50] presented pyrolytic-carbon interdigitated microelectrodes manufactured using UV photolithography techniques, measuring the smallest achievable trench widths in the lower-micron range and pointing out that improvements in the process could yield better yields/products. However, for startups, the primary barriers are economic rather than scientific. Microfabrication concentrates the cost early in the development cycle through high fixed costs and high recurring costs. A fabrication review focused on microfluidic/electrochemical prototyping notes that cleanroom-associated tools span from approximately USD 10,000 to the million-dollar scale, and that cleanroom dependence increases both the cost and operational complexity, motivating cleanroom-free or hybrid prototyping routes for early-stage development [51]. In practice, even when a startup does not build its own cleanroom, shared nanofabrication facilities frequently bill cleanroom access and key tools on an hourly basis, so multi-step process flows translate directly into cash burn during iterative design cycles. Publicly posted user-fee schedules at university nanofabrication facilities illustrate this recurring cost structure, with hourly cleanroom rates and higher external/startup pricing that can materially increase the cost of repeated prototype iterations. Such a high degree of precision normally leads to less variation from sensor to sensor and possibly better analysis [52]. The core argument against microfabrication is its cost and complexity. It demands a clean room setup and expensive capital equipment with long multi-stage process times so the per unit cost far exceeds R2R printing [51,53]. While those in favor say the costs can be amortized across extremely high production volumes, the initial cost as well as operating costs remains prohibitive for many startups and in cases where an extremely low cost is desired [54].

Additive manufacturing approaches are an increasingly visible third pathway for electrode fabrication, especially when a rapid design iteration, unconventional geometries, or integrated structures are required. Unlike R2R printing, which excels at planar throughput, and unlike wafer microfabrication, which excels at sub-micron fidelity, 3D printing can create non-planar conductive architectures and customized form factors with short iteration cycles. Recent reviews summarize how 3D-printed electrochemical sensors can be produced using multiple printer modalities and conductive materials, but they also emphasize that the scale-up bottleneck often shifts from patterning to materials qualification, post-processing control, and the need for standardized in-line metrology [55]. Quantitatively, literature examples report that 3D-printed electrode transducers can achieve very low inter-device variability in controlled studies (e.g., ~0.2% RSD in sensitivity) and can be costed at the level of ~0.11 USD per electrode under lab-scale assumptions; however, these values should be interpreted cautiously because printer-to-printer variation, filament/ink lot variability, and surface activation steps can become dominant error sources without production-grade control plans [56]. In the revised manufacturing comparison (Table 2), we therefore position additive manufacturing as a strong candidate for prototyping-to-pilot translation and for niche products needing 3D architectures, rather than a drop-in replacement for mature R2R strip manufacturing.

**Table 2:** Comparative analysis of scale-up manufacturing paradigms for electrochemical biosensors.

Parameter	Roll-to-Roll Printing (Screen/ Gravure/Inkjet)	Wafer Microfabrication (Photolithography/Thin Films)	Additive Manufacturing (3D Printing/Direct-Write)	How to Interpret for Scale-up and Regulation	Key Literature Anchors
Typical manufacturing intent	Mass disposable, continuous web	High-precision arrays, integration, multiplexing	Rapid iteration; complex 3D features; niche manufacturing	Matching “manufacturing intent” to TPP reduces rework and late-stage quality surprises	Screen-printing vs. additive manufacturing overview [15]
Throughput	~1–50 m/min web speed	Wafer-batch (100–1000s dies/wafer; batch tools)	Minutes per part (printer- and geometry-dependent)	Throughput determines feasible QC strategy (100% in-line vs. sampling)	Lithographic scalability and throughput framing [57]
Feature size/resolution	Typically ~50–200 µm, finer for some inkjet/direct-write	Sub-µm to few µm routinely	~50–500 µm typical for many low-cost printers; finer with specialized direct-write	Resolution matters when sensing depends on diffusion length scales, field confinement, or dense arrays	Microfabrication reproducibility/fidelity emphasized in wafer approaches [58]
Per-unit cost (materials + fabrication, indicative)	Low: often < $0.20/unit at very high volume; glucose strips reported 5–15 cents/strip in mature production [40]	Moderate–high: strongly volume- and die-size-dependent; often > $1/unit at low–mid volume, potentially < $1/unit only when yields and volume are high and design is wafer-efficient	Currently moderate: often > $0.10–$1+/unit depending on print time/material; one report estimated ~$0.11 per electrode for a 3D-printed transducer [26]	We now define “low cost” as <$0.20/unit for disposables, “moderate” as $0.20–$2, and “high” as >$2, to help readers map techniques to business models	Glucose strip cost/yield anchor [40]; 3D-printed cost estimate [26]
Batch-to-batch reproducibility (quantified metric)	Often ~2–5% RSD for redox-probe response in well-controlled batches (example reported < 4.5% RSD for a batch against a standard redox probe) [59]; mature strip manufacturing can reach very low defect rates (<0.1%) [40]	Frequently ≤ 2% RSD reported for device consistency in microfabricated sensors (example: 1.3% RSD in a microfabricated electrode-based sensor consistency test) [60]	Can be excellent in controlled studies (example: ~0.2% RSD for sensitivity reported for a 3D-printed transducer), but may degrade with printer/material variability and post-processing if not controlled [26]	We now define reproducibility bands as excellent ≤ 2% RSD, good 2–5%, moderate 5–10%, and challenging > 10%, because regulatory robustness depends on predictable drift and tight lot release criteria	SPE batch RSD example [59]; microfabricated consistency example [60]; 3D printed RSD example
Quality control maturity for regulated production	Strong for web processes (in-line vision/metrology common); bioreagent deposition can dominate variability	Strong for geometry/films (semiconductor inspection toolchain); biofunctionalization still critical	Emerging; QC and materials qualification often less standardized	Regulatory risk is increasingly a function of QC observability, not only geometry	Additive vs. screen printing manufacturing perspective [15]; 3D-printed sensor review [55]

Finally, manufacturing choice is not a binary decision; the most appropriate route is determined by the TPP. For a mass-market, single-analyte disposable sensor such as a glucose strip, R2R printing remains the most economical option at very high volumes [61]. In contrast, complex multi-analyte implantable sensors or tightly integrated microfluidic lab-on-a-chip systems often rely on microfabrication because geometric tolerance, alignment, and inter-device uniformity directly constrain performance and reliability [62,63]. Between these endpoints, hybrid manufacturing is increasingly used because it assigns each unit operation to the process class that controls it best: R2R methods provide the rapid, low-cost deposition of large-area layers and connectivity across long web lengths, while inkjet/aerosol jet printing is reserved for the localized, digitally metered deposition of high-value functional materials to reduce waste and tighten dose control at the sensing area. This division is especially relevant because the biofunctional layer is frequently a major source of lot-to-lot variability and, therefore, a disproportionate driver of quality risk. A representative hybrid pathway is to fabricate the electrode backplane at scale using R2R, then use inkjet printing to functionalize only the working-electrode region via layer-by-layer deposition and crosslinking chemistries to improve the uniformity and reproducibility of the biological layer. In the literature, a closely related hybrid printing strategy has also been demonstrated by combining the inkjet printing of fine Au electrode features with the rotogravure printing of an enzyme-containing active layer, while quantifying the residual enzyme activity after formulation and after the gravure step, illustrating both the opportunity and the need to manage bioactivity losses during scale-compatible deposition [37,64,65]. Regardless of the specific hybrid route, a QMS with both in-line and end-of-line controls is necessary so that the scaled product continues to meet the performance criteria established during development [61]. Although high-level comparisons of manufacturing paradigms are useful, what determines if something is commercially viable is how well real-world defects can be detected, controlled, and mitigated at high volumes. Table 3 fills the gap and maps out each common manufacturing defect to its function impact and corresponding QC strategy. This table points out a key and frequently overlooked fact: lots of really bad things happening to equipment in the wild do not happen because the fabrication was really, really wrong—they happen because there are few changes that sneak past what people usually test at the end of the line.

Printing-based workflow defect types, like the thickness non-uniformity, nozzle clogging, or coffee-ring formation, translate directly into a variable electroactive surface area and variable local current density, amplifying the sensor-to-sensor dispersion. As shown in Table 3, these risks require in-line optical/metrological control rather than destructive batch sampling only. On the other hand, microfabrication defects are usually geometric or alignment-related, which are already detectable by traditional semiconductor inspection tools before going into production, and this, therefore, enables more confidence in the wafer-level yield. Interestingly enough, Table 3 elevates bioreagent immobilization to a transmanufacturing paradigm cross-cutting failure mode. The variability in enzyme activity retention, adhesion, or dispensing volume often drives the most variability in scaled biosensors even when electrode fabrication is tightly controlled. And this strengthens the argument that biosensor production, which comes with quality assurance requirements, needs to go much further than just to see the physical resemblance—it must care for the biochemical workability, stabilities, aging performance, etc. Everything needs to go together, and that is what a lot of lab experiments at the lab scale will mostly overlook, but it will still be vital to make sure you get the regulatory permits as well as being able to stay in the market over a while, of course.

## 4. Regulatory and Quality Considerations: Navigating the Global Landscape

Even if there is a robust supply chain and a manufacturing process that is just right, an electrochemical biosensor cannot get to market unless it gets past the huge obstacle of getting approved by the regulators [74]. For devices that will be used in clinical diagnostics, we need to navigate a maze that is complex, costly, and time-consuming of national and international rules to guarantee the safety of patients and the effectiveness of the device [75,76]. A start-up, academic-spinout’s failure to build regulation into the development plan at the outset is a common, often fatal, omission [77]. The three most important regulatory areas for medical devices include the U.S. (FDA), EU (CE mark under IVDRE), and, increasingly, China (NMPA). Though they have the same goal, their exact ways, needs, and ideas do differ greatly [76].

In the United States, the FDA regulates medical devices using a risk-based classification system (Class I, II, and III) that is explicitly tied to the level of regulatory controls needed to provide a reasonable assurance of safety and effectiveness. Class I devices are generally subject to ‘general controls’, Class II devices are subject to general controls plus ‘special controls’, and Class III devices are those for which general and special controls are insufficient and, thus, typically require PMA [78]. Most non-implantable electrochemical IVD/POC biosensors fall into Class II, where the dominant premarket route is the 510(k) Premarket Notification. A 510(k) submission is a comparative pathway: the sponsor must demonstrate that the new device is ‘substantially equivalent’ to a legally marketed predicate device in terms of intended use and technological characteristics. Accordingly, the typical 510(k) evidence package is structured around analytical performance testing, method comparison, and risk controls sufficient to satisfy general and special controls, with clinical data required only when bench/analytical testing and predicate comparisons are insufficient to support a substantial equivalence [79]. By contrast, Class III devices (e.g., life-sustaining/life-supporting devices or those presenting a potential unreasonable risk) generally require PMA, which is an affirmative, stand-alone demonstration of safety and effectiveness rather than a predicate comparison. PMA submissions, therefore, tend to be more data-intensive and include extensive nonclinical and clinical evidence, detailed manufacturing information, and FDA inspectional oversight. In addition, PMA applications follow a different documentation structure and create a more stringent lifecycle for post-approval changes [80]. For electrochemical biosensors, this distinction is commercially consequential: a Class II/510(k) strategy often favors predicate-anchored, incrementally comparable designs (and a faster iteration within the bounds of substantial equivalence), whereas a Class III/PMA strategy typically increases the clinical evidence and quality-system burden but can enable higher-risk indications (e.g., long-term/implantable monitoring) with more extensive approved claims and tighter postmarket change-control. A useful “real-world” illustration of the ways in which electrochemical platforms are put to work in the 510(k) paradigm is Abbott’s i-STAT cartridge ecosystem (an archetypal cartridge-based electrochemical sensing translated into routine care) [80]. For example, the i-STAT TBI Plasma cartridge was cleared by 510(k) as an adjunct, rapid plasma test that runs on a previously cleared i-STAT analyzer; the publicly available FDA review documentation is clear that the regulatory argument is based on a tightly defined intended use, method comparison, and analytical performance evidence, rather than novel and open-ended claims of novelty, even with a technologically sophisticated underlying measurement. In practice, this “predicate-anchored” logic incentivizes small changes while structurally disincentivizing large changes that would make things uncomparable and make a sponsor go from 510(k) to PMA.

A big point of argument in both schools and factories is that people think the FDA’s review process is too strict and hard to guess, so they say it stops making new things and makes sick people wait longer to use them [81,82]. Obtaining FDA clearance or approval is difficult; there is a lot of analysis validation data required, such as precision, accuracy, linearity, interference, and stability study, as well as good clinical trials to prove performance in the intended patients [83]. But the counterpoint is that “stringency” is not just bureaucratic friction, but rather a forcing function to make early material highly susceptible to manufacturability and long-term field performance, and design choices. The PMA experience of Senseonics’ implantable CGM system (Eversense) shows this tradeoff. Senseonics got the CE mark in Europe for the Eversense system beforehand (under the pre-IVDR regime) [84], but getting to the U.S. market needs PMA-level evidence and life cycle controls, and FDA PMA records publicly show a regulated path that cannot be separated from clinical study expectations and ongoing supplement-based change control after initial approval [85]. From a strategy standpoint, the implication for electrochemical biosensors is that regulatory classification is not just a “review hurdle,” but a driver of what kind of design iteration can remain economically justifiable post-launch.

Recently, the European Union has been going through a large amount of regulatory change, changing from the In Vitro Diagnostic Directive (IVDD) to the much more strict In Vitro Diagnostic Regulation (IVDR 2017/746) [86]. One important shift philosophically with the IVDR is going from a list-based approach to a risk-based classification (A,B,C,D) so it lines up better with the FDA model [87]. And, so, that changed the level of scrutiny that most IVDs would have to go through. Under the IVDD, a very high percentage of manufacturers were able to self-certify. According to the IVDR, it is believed that more than 80% of IVDs, including many electrochemical biosensors, now require some involvement by a third-party conformity assessment body known as a Notified Body to obtain a CE mark [86]. There is a whole different emphasis now on the IVDR—it is going to have to put in place a much broader emphasis on proof, with a robust Performance Evaluation Report, PER. That will include information about the scientific validity and then analytical performance and clinical performance over the whole life cycle of the device, which would make collecting data and recording much stricter, quite troublesome, and even harder for the smaller ones. The EU’s model is different from the FDA in more ways than just the structure: the capacity and variability of Notified Bodies becoming a bottleneck independent of science merit is a worry serious enough to see EU institutions push out transitional timetables to mitigate the risk of test shortages and markets shutting down. Electrochemical biosensors run into practical self-contradiction: the cost-per-test can be low, but also alarmingly high to certify and keep compliant after real-world performance oversight and the full cost-of-periodic-technical-documentation-updates kicks in.

And China’s NMPA has become another important regulatory body, showing how healthcare is becoming more important in China, and the growing importance of China as a place to make new medical devices [88]. The NMPA employs an analogue three-tier system to the FDA’s. For foreign manufacturers, one of the big requirements for getting through the NMPA is that all the clinical trials you want to register need to happen in China—all or part of it. This can make the time and money needed for approval go way up [89]. But the NMPA also created quicker ways for new medical devices that are better than older ones, if they are shown to be a really big help for patients, which could make it go faster [88,89]. A specific example is MicroTech Medical’s continuous glucose monitoring system (AiDEX G7). MicroTech publicly declared the NMPA registration approval (4 November 2021) and presented it as an innovative CGM system comprising the sensor, transmitter, receiver/software, and accessories. The company then placed emphasis on the “calibration-free” real-time CGM performance as a clinical differentiator for the China market in the subsequent corporate reporting along with the regulatory burden associated with Class III active devices. From a critic, this shows a very different NMPA dynamic: the regulator can be both demanding and industrial-policy-aligned, such that regulatory success is usually more about being in the right local execution environment than just having a good sensor.

As shown in Table 4, regulatory pathways across the United States, the EU, and China converge on a risk-based philosophy, but they differ in how they structure and interrogate the performance evidence. In the U.S., the FDA review is typically organized around a tightly defined intended use and (for many Class II pathways) substantial equivalence, with the submission emphasizing analytical performance and method comparison data sufficient to support the claimed use and labeling limitations (performance characteristics such as precision, detection capability, and analytical specificity/interference are commonly expected elements of the analytical validation package). Under the EU IVDR (Regulation (EU) 2017/746), performance evidence is framed as a life-cycle performance evaluation that must integrate scientific validity, analytical performance, and clinical performance, aligned to the General Safety and Performance Requirements and documented in structured performance evaluation documentation. In China, the NMPA’s IVD governance similarly uses a risk-based classification and places strong procedural weight on defined product technical requirements, registration testing, and China-context execution (including local evidence generation expectations for certain products), so that the analytical performance claims are closely coupled to formal testing and compliance pathways. From a translation standpoint, this means the regulatory strategy is not a late-stage checklist; it is an early system design constraint that shapes the assay architecture, manufacturing controls, and the scale and type of evidence needed to sustain post-market changes.

All regulatory submissions are supported by a solid QMS. Adherence to ISO 13485:2016 has effectively become a global baseline because it structures design controls, risk management (e.g., ISO 14971 [96]), supplier controls, process validation, and post-market surveillance so that performance claims remain traceable to controlled processes. For regulatory submission, the key analytical performance metrics are not just “good-to-have” descriptors; they are the quantitative evidence used to justify the device’s intended use and limitations in labeling and technical documentation, and they often appear explicitly as required performance characteristics (e.g., precision, limits of detection, and interference controls). In practice, sponsors and reviewers frequently operationalize these concepts using consensus measurement standards (e.g., CLSI EP05 for precision, EP17 for detection capability, and EP07 for interference testing), because they provide reproducible study designs and reporting conventions suitable for dossier review. Under the EU IVDR, the same analytical metrics are embedded within the General Safety and Performance Requirements and are assembled as part of the device’s performance evaluation (scientific validity, analytical performance, and clinical performance) rather than treated as standalone “lab metrics.” In China, NMPA registration similarly ties performance claims to defined product technical requirements and formal registration testing performed through designated testing pathways, reinforcing that these metrics become auditable acceptance criteria rather than one-time academic demonstrations.

In addition to single-lot analytical validation, regulators and notified bodies are concerned with whether performance crosses manufacturing variability, particularly reagent and bioreagent lots. For electrochemical biosensors that incorporate antibodies/enzymes in strips, cartridges, or immobilized layers, lot-to-lot variation is a recognized pathway to bias and imprecision that can affect conformity to accuracy standards and can trigger post-market corrective actions if not controlled. The finished-product experience in glucose monitoring demonstrates that the strip lot-to-lot differences can be large enough to change whether an SMBG system meets ISO criteria on a per-lot basis, highlighting that ‘lot’ is a compliance unit, not only a manufacturing detail [97]. Consistent with this logic, FDA review packages for cartridge-based electrochemical testing explicitly evaluate precision across multiple cartridge lots, reflecting the expectation that analytical performance evidence must cover lot variability rather than a single optimized batch. Accordingly, an ISO 13485-aligned quality system should treat bioreagent lots as controlled inputs with defined acceptance criteria, lot-bridging protocols, and traceable release decisions (functional activity assays plus electrochemical lot benchmarking), so that the analytical claims summarized in Table 5 are demonstrably robust to routine lot changes. The performance metrics summarized map directly to the failure modes most likely to preclude real-world clinical utility. More importantly, these are not independent silos: for example, very aggressive LOD optimization via nanostructured electrodes or signal amplification can make it so the system is unstable with respect to precision or long-term stability, such that aging and interference studies would be even more burdensome. Thus, Table 5 points out an important conflict in biosensor translation: academic displays usually stress sensitivity or newness, but regulators value consistent, reproducible performance across real clinical situations. From a quality system perspective, these metrics also become design inputs and acceptance criteria in an ISO 13485-compliant workflow and tie early design decisions for assays back to downstream verification, validation, and post-market surveillance obligations. Embedding Table 5 at this point in the narrative makes the case that regulatory expectations are not an outside imposition but a formalization of good measurement science that rewards the tradeoff of a good result in disciplined contexts, rather than peak laboratory performance.

To reduce the gap between ‘reporting an analytical parameter’ and ‘establishing it in a form reusable for regulatory evidence,’ it is helpful to state what each commonly cited CLSI evaluation protocol actually requires in practice. EP05 operationalizes precision as a multi-day, multi-run replicated design (the commonly used 20 × 2 × 2 experiment) specifically intended to separate within-run repeatability from within-laboratory sources such as day and run effects. EP17 defines the detection capability as a hierarchy (LOB, LOD, and LOQ) and provides explicit statistical estimators for LOB and LOD that depend on replicated blank and low-level samples, plus an LOQ concept tied to an accuracy goal rather than a single instrument noise statistic. EP09 formalizes method comparison and bias estimation using split patient samples across the measuring interval and explicitly links the study design to the defensibility of bias claims; in EP09-A3, validation/claim verification is recommended to use at least 100 patient samples collected across multiple days. EP06 frames linearity as allowable nonlinearity across a stated interval using multiple concentration levels and replicated measurements, supporting a reportable range defined by a criterion rather than by visual inspection alone. EP07 provides a structured approach for screening and quantifying interference effects, including defining what counts as medically significant bias and documenting interferent-specific claims. EP25 extends the same logic to stability claims, requiring that shelf-life and in-use statements be supported by planned studies with acceptance criteria linked to the device’s analytical claims. In biosensor research practice, these protocol elements can be adopted in a ‘regulatory-ready’ way without waiting for a full submission stage; for example, EP17-style replicate blank and low-level designs are already used in some biosensor performance studies to compute LOB/LOD/B explicitly rather than reporting LOD as S/N-based extrapolations. Similarly, regulator-facing reviews of electrochemical glucose systems illustrate how linearity and matrix challenges are structured with multiple concentration levels and hematocrit strata, emphasizing the preplanned coverage of clinically relevant conditions rather than only idealized buffer tests.

The digital health regulation for electrochemical biosensors, SaMD and AI/ML, and the cybersecurity of commercially relevant electrochemical biosensors are no longer ‘sensor-only’ products; they are cyber-physical systems in which software performs safety-relevant medical functions. In this review, we use the internationally convergent terminology of Software as a Medical Device frame this shift. IMDRF defines SaMD as software intended for one or more medical purposes that performs these purposes without being part of a hardware medical device [1]. This definition becomes relevant when electrochemical signals are processed, interpreted, or transformed into clinical recommendations by software that can be decoupled from the sensing hardware (e.g., app- or cloud-based analytics). Even when software remains integrated with hardware, modern regulatory practice treats many ‘software functions’ as regulated elements requiring lifecycle controls, traceability, verification/validation, and post-market change management consistent with medical device quality systems. From a commercialization standpoint, the key regulatory challenge is that algorithmic calibration and compensation can change clinical performance as strongly as changes in electrode materials or biorecognition chemistry. Therefore, evidence packages for electrochemical biosensors that rely on advanced signal processing should explicitly justify how the software was developed, validated, and maintained across the total product lifecycle. International standards provide a structured implementation route: IEC 62304 specifies medical device software lifecycle processes, emphasizing risk-based development and maintenance requirements; complementary health software requirements and cybersecurity lifecycle expectations are increasingly recognized by regulators and procurement systems for connected medical products.

AI/ML-enabled functions further expand the compliance surface area because the model performance can degrade or shift under real-world deployment (data drift, population shifts, and sensor aging), and because updates may change clinical behavior. The FDA has formalized a lifecycle-oriented oversight direction for AI/ML-based medical software, including the AI/ML-Based SaMD Action Plan and subsequent guidance mechanisms aimed at enabling iterative improvement while maintaining a reasonable assurance of safety and effectiveness. A central concept is the Predetermined Change Control Plan (PCCP), which describes planned modifications and how they will be assessed; this is particularly relevant to biosensor products where algorithm updates may be frequent (e.g., improved calibration models or artifact rejection). Published analyses underscore the scale of this regulatory issue: by the end of 2024, more than one thousand AI/ML-enabled medical devices had been authorized through FDA pathways, motivating clearer expectations for safe post-market updating. In the European Union, IVDR implementation should be interpreted together with MDR/IVDR software guidance (e.g., MDCG documents), because software that drives clinical decisions or influences the use of a device can affect qualification, classification, and conformity assessment obligations. For electrochemical biosensors that integrate mobile applications, cloud connectivity, or decision support, developers should anticipate that cybersecurity, data governance, and software change control will be evaluated as part of the technical documentation and post-market surveillance plan, not as an optional ‘IT feature.’ Collectively, these digital-health requirements reinforce the paper’s central argument: the translational barrier is a system-level problem, and successful scale-up requires the co-design of the wet-chemistry assay, manufacturing controls, and software/regulatory lifecycle strategy from the earliest development stages.

## 5. Case Studies and Future Perspectives

To illustrate how the manufacturing strategy can determine commercial outcomes, the personal glucose meter provides the canonical example in electrochemical biosensing. Its success was enabled not only by robust enzymatic electrochemistry, but by the fact that the disposable strip could be manufactured at an extreme scale with a high yield and tight process control using high-throughput thick-film screen printing and roll-based converting. In this workflow, the electrode and reagent layers are deposited additively and sequentially on polymer films (typically including a carbon working electrode and an Ag/AgCl reference/counter electrode, followed by dielectric insulation and printed or coated reagent/mediator chemistry), then dried/cured in continuous lines before lamination with spacers/adhesives, slitting, and high-speed die cutting into individual strips. This manufacturing paradigm minimizes material waste, supports automation and in-line inspection (registration, layer thickness, and electrical continuity), and enables consistent electrochemical geometry and reagent loading across very large production runs. As a result, the glucose-strip industry reached production volumes on the order of billions of electrochemical strips per year, while maintaining a low estimated manufacturing cost (reported as ~5–15 cents per strip) and very low defect rates (<0.1%), performance levels that are difficult to achieve with low-throughput laboratory fabrication methods [40]. A concrete example of how printing supports controlled electrochemical architectures in commercial products is the FreeStyle strip design described as a printed parallel-plate coulometer with an approximately 50 μm gap between facing printed electrodes, illustrating that high-throughput printing can repeatedly realize not only a low cost but also reproducible micro-scale structures relevant to measurement performance [108]. Economically, these manufacturing capabilities enabled a high-volume consumables model in which the meter could be priced aggressively while sustainable revenue came from recurring strip purchases; operationally, they required the stable sourcing of enzymes (e.g., glucose oxidase or dehydrogenase), mediator chemistries, and conductive inks, coupled with lot-qualification and release testing to protect strip-to-strip consistency. Overall, the glucose meter demonstrates that the ‘commercial translation’ advantage of screen printing is not merely faster prototyping, but the ability to industrialize a multilayer electrochemical device into a regulated, high-yield commodity at massive scale [109].

However, the glucose-strip “template” is less transferable when we go from single-analyte, short-duration, capillary-blood testing to continuous sensing, multi-component integration, and digital ecosystems. A first instructive bridge case is the hospital-grade glucose biosensor, represented by Nova Biomedical’s StatStrip Glucose Hospital Meter System, which received FDA clearance for multi-patient, point-of-care use across acuity, including critically ill patients [110]. In comparison to consumer strips, the regulatory and quality burden is moving from alleviating problems with consumers to make things work with less trouble in the world around them. and moving towards making the regulation and making sure safe operations can happen when it is complicated. It pushes them towards more restrictive incoming quality controls on membranes, mediators, and strip fabrication tolerances, and towards cybersecurity and connectivity in next-generation platforms. Strategically, it is the same electrochemical reaction, but different commercialization constraints when the use environment and risk posture change.

A second case, showing another commercialization logic, is coagulation self-testing like Roche’s CoaguChek XS (amperometric pt/INR determination with single-use strip) [111]. Unlike with glucose, we are not making the case primarily for ultra-high-frequency everyday testing on a really, really big population. We are really making the case for risk reduction and therapy optimization in a much more constrained subset. Thus, the result is that, though the device may still follow a consumable-strip model, it makes a difference in the market scale and the pricing power of the supplier, and also with the supply chain sensitivities: the strips are embedded with thromboplastin and other reagents, which will be affected by the stability and the consistency of each batch, and they can affect the clinical decisions directly. This case underscores a major commercialization lesson: electrochem disposables are not, by default, “low-risk commodities”; assays closer to clinical decision thresholds need stronger reagent characterizations, stability protocols, and post-market surveillances for regulatory comfort and reimbursement buy-in. A third clinically mature case family is cartridge-based point-of-care testing that integrates microfluidics with electrochemical sensor arrays, exemplified by Abbott’s i-STAT platform. The i-STAT cartridge family includes potentiometric and amperometric sensing elements and, for multi-analyte chemistry panels such as CHEM8+ [112], the cartridge is intended for the quantification of electrolytes, metabolites (including glucose), and hematocrit in whole blood in point-of-care or clinical laboratory settings, with multiple cartridges cleared through the FDA 510(k) pathway [113]. What changes versus consumer glucose strips is not the existence of electrochemical transduction, but the risk posture of the use case and, therefore, the tolerance for manufacturing variation. For OTC self-monitoring glucose strips, commercialization is dominated by the very high volume, cost compression, and lot-release strategies that must still meet self-testing accuracy expectations; the primary manufacturing burden is maintaining the process capability and reproducibility at scale (e.g., screen-print thickness control, enzyme deposition uniformity, and statistically controlled lot-to-lot performance). For hospital-grade systems such as i-STAT, the cartridge is used in acute-care workflows where multi-analyte results may drive immediate clinical decisions, so the acceptable failure modes narrow and the compliance surface expands: incoming material controls extend to multilayer polymer films, adhesives, microfluidic laminates, calibrant solutions, and sensor membranes; traceability and change control become more stringent; and several critical processes require formal process validation because end-product testing cannot fully verify the quality (for example, microfluidic sealing integrity and fluidic path reliability). In this setting, ‘scale-up manufacturing’ is less about roll-to-roll throughput and more about validated assembly windows, seal integrity assurance, drift control, and stability across distribution and storage, because a single cartridge failure is operationally and clinically consequential rather than a marginal yield loss.

A closely related fourth case is Siemens Healthineers’ epoc Blood Analysis System, which was cleared through the FDA 510(k) [114] and uses single-use test cards that incorporate multiple sensors for blood gases, electrolytes, and metabolites. Compared with i-STAT, epoc shows commercialization enabled by room-temperature-stable consumables and simplified workflow, which reduces the cold chain complexity and material, risking global distribution. However, this comes at a cost: stabilizing the chemistry, making sure that the sensor behaves consistently over a temperature range can make the materials and the processes more complicated. This will require a strong process validation and supplier qualification. In critical-care POC markets, such reliability engineering can be a very effective commercialization moat—but it also raises a barrier for an academic spin-out underestimating how fast even small material micro-variations translate into clinical outliers.

But compare that with the new area of wearable multiplexed electrochemical biosensors which continuously monitor things like lactate or cortisol or electrolytes in your sweat or interstitial fluid. From a supply-chain perspective, the bill of materials expands beyond electrodes and bioreagents to include medical-grade skin adhesives, barrier films, hydrogel/interface layers, microfluidic laminates, and miniaturized electronics (ASIC/BLE components and batteries). Each of these inputs can become a dominant failure mode: for example, adhesive and laminate suppliers must provide tightly controlled thickness, water-vapor transmission, and biocompatibility documentation to maintain both wear comfort and signal stability, while microfluidic materials must be consistent enough to avoid a channel-to-channel flow imbalance that distorts time-stamped sampling. These constraints are amplified in multiplexing, because the lot drift in any single sensing element can propagate into cross-analyte inconsistency and undermine the product’s central value proposition. In manufacturing, multiplex sweat patches shift the scale-up risk from printing alone to system integration and yield management across heterogeneous unit operations: the multilayer alignment/lamination of microfluidics, robust sealing to prevent leakage/evaporation, the repeatable formation of reference electrodes, and the mitigation of analyte cross-talk via fluidic isolation. The prior literature on microfluidic sweat patches emphasizes that sweat-rate variability and sampling artifacts are not minor laboratory nuisances but core engineering variables that must be measured and controlled at the product level; consequently, manufacturable designs increasingly integrate sweat-rate sensing and engineered microfluidic routing to preserve temporal fidelity [115]. A practical translation-relevant signal is that some microfluidic sweat patches have been explicitly designed for high-volume fabrication via roll-to-roll processes, indicating that commercialization depends on manufacturable architectures rather than peak analytical sensitivity alone. Regulatory and quality strategy is also uniquely complicated relative to traditional sensors. First, classification often hinges on claims: general wellness positioning may reduce premarket burden, whereas disease-related claims can trigger medical device oversight and higher evidence expectations [116]. Second, multiplex patches typically generate continuous, software-mediated outputs, which expands the compliance scope to sensor-based digital health technology and software lifecycle controls, and it raises requirements for cybersecurity, update control, and human-factors validation because interpretation occurs in real-world, unsupervised use. Recent FDA policy documents reinforce that the boundary between wellness and medical claims is central for wearables, while also highlighting that FDA’s framing of sensor-based digital health devices explicitly includes wearable patches, which increases the practical importance of early intended-use decisions for commercialization timelines and quality-system design. Collectively, these factors mean that the ‘commercial bottleneck’ for multiplex sweat patches is rarely electrode fabrication alone; it is the coupled control of materials, microfluidic sampling integrity, multi-analyte calibration/algorithm governance, and digital-regulatory compliance as a single product system.

However, the wearable ‘multiplex’ should not be treated as one category: continuous glucose monitoring (CGM) is already a mature commercialization pathway with constraints that differ from both traditional fingerstick meters and emerging sweat patches. Abbott’s FreeStyle Libre family (e.g., Libre 2/3) and Dexcom systems (e.g., G6 and G7) illustrate that CGM product success is driven by systems engineering more than electrode chemistry alone. In CGM, the electrode is only one element inside a coupled measurement-and-decision system, where real-world accuracy is co-determined by membrane mass-transport control, enzyme and mediator stability, sensor–tissue interface biology (biofouling/foreign-body response), insertion mechanics, and continuous algorithmic calibration and filtering that converts a noisy interstitial signal into actionable glucose estimates. Long-term in vivo performance is limited by tissue responses and material–tissue interactions that alter the diffusion and sensitivity over time, which is why commercial CGM architectures invest heavily in outer membranes/coatings and biocompatible packaging rather than only optimizing the catalytic current in vitro [117]. This ‘system’ framing is also visible in how CGM accuracy is defined and improved. Accuracy is typically assessed against reference blood glucose using metrics such as the mean absolute relative difference (MARD), and the literature emphasizes that the measurement performance depends strongly on calibration practices and signal-processing algorithms rather than sensor chemistry alone [118]. In silico and clinical discussions of non-adjunct CGM use further highlight that an accuracy threshold is a system property, not a single-material property. Commercial CGM therefore succeeds when manufacturers can (i) lock down manufacturing variation across lots, while simultaneously (ii) operating a controlled software lifecycle for calibration and data interpretation within a regulated quality system. The practical consequence is that ‘electrode chemistry optimization’ is necessary but not sufficient: interference susceptibility and drift must be managed at the system level, including explicit interference evaluation and labeling, and, for connected CGM, cybersecurity and software update controls [119]. Human factors and insertion usability are equally commercialization-critical because poor insertion or wear adherence generates signal artifacts and early failures that cannot be solved electrochemically; for example, the CGM design reports and usability studies emphasize applicator design and user-task performance as safety-relevant engineering outputs [120]. Finally, the CGM regulatory structure directly encodes this systems requirement: the FDA’s integrated CGM (iCGM) classification and special controls emphasize design verification/validation across accuracy, reliability, and interoperability, reinforcing that CGM is regulated and commercialized as an integrated system (sensor + transmitter + software), not as an isolated electrode.

A sixth case complicates the wearable narrative: Medtronic’s invasive glucose sensors, like the Guardian Sensor (3), which were cleared via PMA supplements [121], and later systems approvals with broader indications using newer sensors (Guardian 4 with auto insulin delivery systems). Compared with 510(k)—routed glucose meters, and PMA—governed CGM components raise evidentiary demands and change-control friction. This results in a commercialization tradeoff—a higher regulatory burden may slow down iterations but could also result in stronger clinical claims and more integration with closed-loop insulin delivery. Strategically, this is why so many startups struggle with “continuous wearable biosensing”—it takes more than fabrication, and you need the capital stamina to maintain a quality system, create the clinical evidence, and iteratively submit to the regulators across multiple product generations.

Looking ahead, several trends will shape the future commercialization of electrochemical biosensors. First, there is a strong push toward sustainable manufacturing. Single-use plastic disposables are convenient but raise clear environmental concerns. Accordingly, research and development is increasingly exploring biodegradable materials such as paper and modified cellulose, and greener synthesis routes for functional nanomaterials to reduce the lifecycle impact of manufacturing and disposal [118]. However, for clinical diagnostics, sustainability targets must be reconciled with the stability and shelf-life evidence required for regulated claims. In practice, adopting biodegradable or bio-based substrates can introduce additional degradation pathways and variability risks that are less dominant in conventional petrochemical polymers, including humidity-driven swelling and dimensional drift in cellulose-based webs, changes in surface wettability that alter reagent adsorption and drying behavior, increased sensitivity to microbial contamination, and, for degradable polymers, hydrolytic aging or additive migration that can affect the mechanical integrity, electrode adhesion, and analytical drift. These risks do not preclude sustainable materials, but they shift the design space toward ‘sustainable-by-design’ architectures that are coupled to stability engineering, for example, barrier layers or encapsulation stacks with quantified moisture and oxygen transmission performance, and packaging configurations selected to control humidity and oxygen exposure during distribution and storage. Crucially, any sustainability-motivated material change must be validated through stability-claim studies that are acceptance-criteria-driven and analytically anchored (e.g., allowable drift in bias/precision, control recovery, and calibration slope), supported by real-time stability data and, where justified, accelerated aging that is subsequently confirmed by real-time studies in the intended packaging configuration, consistent with established stability-claim frameworks for IVD products (e.g., CLSI EP25 and ISO 23640 [96]) and accelerated-aging methodologies commonly used for medical device packaging and materials (e.g., ASTM F1980).

Secondly, Industry 4.0 tools can directly mitigate the inconsistency that limits large-scale rotated biosensors by turning the ‘hidden’ process drift into measurable variables and then correcting it in real time. In R2R printing, small time-varying disturbances in web tension/speed, nip force, ink rheology, and drying conditions propagate into overlay/registration error, line-width variation, and thickness non-uniformity, which ultimately appear as sensor-to-sensor dispersion in resistance, electroactive area, and calibration slope. A practical Industry 4.0 response is to pair the machine-vision metrology with real-time in-line metrology for thickness and surface uniformity, enabling closed-loop control rather than end-of-line ‘pass/fail’ sampling. Real-time in-line metrology is increasingly recognized as the enabling layer for stabilizing continuous R2R manufacturing because it supports the rapid detection of drift and corrective action before large volumes are produced out of specification [41]. In parallel, a digital twin provides a virtual replica of the R2R line that assimilates sensor streams (tension, speed, temperature, and humidity) and learned models of process dynamics to predict when the quality will deviate, so the control actions can be proactive rather than reactive. For example, in roll-to-roll gravure printing, a digital-twin approach has been demonstrated to predict overlay printing registration error from web tension, nip force, and printing speed, achieving a 77% prediction accuracy for machine-direction error when combined with the camera-based misalignment measurement of printed markers [118]. Similarly, a digital twin for R2R web handling has been shown to predict web-tension signal features with test-set R^2^ values ranging from ~52 to 100% (model-dependent) and to operate with near real-time data transfer (~0.1 s delay), enabling the early detection of abnormal behavior and predictive-maintenance triggers when the observed signals diverge from the predicted ‘healthy’ baseline [118]. For biosensor production, these capabilities matter because they target the manufacturing failure modes that most strongly drive lot-to-lot variability (registration drift, thickness non-uniformity, and defect emergence across long rolls), while also generating traceable, time-stamped quality data streams that can be linked to batch records and process validation evidence under a Quality-by-Design mindset.

And, finally, there are going to be rules as well, and they are going to be changing too. Gradually, but surely, there is a movement towards a world regulation led by groups like the IMDRF (International Medical Device Regulators Forum) to get in line with standards and make the approval procedures less cumbersome from one country to another. Meanwhile, as personalized medicine and digital health expand, regulators are formalizing expectations for adaptive software functions, in algorithms used for calibration, signal processing, and decision support. This is especially important for next-generation electrochemical biosensors where algorithmic compensation can be a primary determinant of clinical performance. In the United States, the FDA has articulated a total product lifecycle orientation for AI/ML-based medical software, including the AI/ML-Based SaMD Action Plan and the subsequent guidance mechanisms intended to support iterative improvements through a PCCP while maintaining a reasonable assurance of safety and effectiveness. In the European context, IVDR/MDR implementation is complemented by MDCG guidance that clarifies the qualification and classification of software, which can materially affect the conformity assessment requirements when the software drives clinical decisions or influences the use of a device. For the commercialization strategy, the implication is that digital functionality should be treated as a regulated subsystem with its own design controls, risk management, verification/validation, cybersecurity engineering, and post-market performance monitoring, rather than as an accessory added after the electrochemical platform is finalized. The U.S. trajectory of formalization of the “integrated CGM” (iCGM) as a class II device type with special controls is clear—regulators are working to create pathways for interoperability and performance standardization that reward manufacturers, but they are also increasing the compliance surface area for device makers [122]. The companies who are going to win out are going to be the companies that are, firstly, not simply companies who are incredibly, extremely, profoundly technologically advanced—they actually have to be advanced in their agility and in their nimbleness, agile and nimble enough for them to not just be able to compete in the changing landscape around them that is going to be full of technology, but also changing due to the regulatory policy landscape out there.

## 6. Conclusions

And this review reveals it is whether or not electrochemical biosensors actually succeed at getting from labs to customers that has much less to do with whether you can get tiny, little improvements in analytical sensitivity first and much more to do with the business being first about dealing with the supply chain’s strength and robustness, scalable manufacturing, and the regulatory–quality aspects. Looking across the literature and case studies that have been looked at, there is always this same kind of pattern where the choices made about what materials to source from, what manufacturing setup to go for, and how to deal with any regulations seem to be very tied up with each other as things to think about at once instead of one after another. Resilient supply chains will require making deliberate tradeoffs between cost efficiency and risk mitigation, especially with respect to biorecognition elements and functional inks, whose lot-to-lot variation directly propagates as analytical drift and regulatory non-compliance. Scale-up manufacturing is demonstrated as a strategic choice for target product profiles: roll-to-roll excels in ultra-high-volume disposables, microfabrication for complex or high-risk applications, and geometric control. Hybrid approaches are becoming more common. Importantly, the manufacturing defects that matter in the lab do not matter at the commercial scale where field failures are driven by those defects. Thus, in-line and end-of-line quality controls that go beyond physical pattern fidelity and include biochemical functionality and stability are necessary. Regulatory classification from a regulatory perspective of comparing the FDA, IVDR, and NMPA pathways is found to show that it is not merely an approval hurdle, but, rather, a development timeline and capital intensity and post-market design flexibility. The reviewed case studies that go from glucose strips to cartridge-based systems to continuous monitoring platforms also demonstrate that the same electrochemical principles can have radically different commercialization constraints based on the use context and risk posture. Collectively, these reinforce a common conclusion: electrochemical biosensors succeed commercially, and are not commercial failures, when the translation problem is treated as a system-level engineering and management challenge rather than as a late-stage add-on to the problem of optimizing laboratory performance. Therefore, future progress will have to be made by getting academic research practices closer to industrial ones—in terms of standardization, manufacturability-aware design, and regulatory-grade validation—so that more innovations can make it across the valley of death into sustainable clinical and societal impact.

## Figures and Tables

**Table 3:** Common manufacturing defects in scaled production and associated QC measures.

Manufacturing Method	Common Defect	Potential Impact on Performance	In-Line QC Method	End-of-Line QC Method	References
Screen Printing	Inconsistent layer thickness, pinholes, smearing	Altered electrode area, variable resistance, short circuits	AOI, laser profilometry	Electrochemical testing (e.g., CV), impedance spectroscopy	[66,67]
Inkjet Printing	Satellite droplets, clogged nozzles (“missing jettle”), “coffee ring” effect	Inconsistent electrode morphology, open circuits, non-uniform bioreagent distribution	Stroboscopic imaging of droplet formation, real-time vision systems	Microscopic inspection, functional testing with analyte	[68,69]
Photolithography	Under- or over-etching, resist adhesion failure, misalignment	Incorrect feature dimensions, delamination, device failure	Critical Dimension Scanning Electron Microscopy (CD-SEM), overlay measurement tools	Wafer-level electrical probing, release testing	[70,71]
Bioreagent Immobilization	Inconsistent dispensing volume, enzyme denaturation, poor adhesion	Variable sensitivity, reduced shelf life, poor reproducibility	Gravimetric analysis (for dispensing), fluorescence-based activity assays	Accelerated aging studies, batch release testing with controls	[72,73]

**Table 4:** Comparative overview of major regulatory pathways for IVD devices.

Feature	U.S. Food and Drug Administration (FDA)	European Union (CE Mark Under IVDR)	China National Medical Products Administration (NMPA)
Governing Regulation	21 CFR Part 820 (QSR), Part 809 (IVDs)	Regulation (EU) 2017/746 (IVDR)	Decree No. 739 (Regulations for the Supervision and Administration of Medical Devices)
Risk Classification	Class I (Low), II (Moderate), III (High)	Class A (Low), B, C, D (High)	Class I (Low), II (Moderate), III (High)
Primary Review Pathway	510(k) Premarket Notification (Class II), Premarket Approval (PMA) (Class III)	Notified Body Conformity Assessment (Class B, C, D), Self-declaration (Class A)	Registration (Class II & III), Filing (Class I)
Key Submission Dossier	510(k) or PMA submission with analytical and clinical data	Technical Documentation, including Performance Evaluation Report (PER)	Registration Dossier with local type-testing and clinical trial data
Clinical Evidence Requirement	Clinical trials often required for PMA; may be required for 510(k) if predicates differ	Mandatory for most classes; continuous lifecycle approach to data collection (PMPF)	Local clinical trials are mandatory for Class II and III devices not on exemption list
Quality System Standard	Quality System Regulation (QSR)	ISO 13485:2016 (harmonized standard)	Good Manufacturing Practice (GMP) guidelines
Key Challenge/ Debate	Perceived as slow and unpredictable, potentially stifling innovation	Shortage of Notified Bodies, increased data burden under IVDR	Requirement for local clinical trials, rapidly evolving regulations
References	[90,91,92]	[92,93,94]	[10,92,95]

**Table 5:** Key analytical performance metrics for regulatory submission of electrochemical biosensors.

Performance Metric	Definition (IUPAC/CLSI Based)	Importance for Clinical Use	Typical Validation Method	References
Precision (Repeatability and Reproducibility)	The closeness of agreement between replicate measurements under stipulated conditions (e.g., same run, different days, different operators/instruments).	Ensures consistent results for patient monitoring and treatment decisions.	CLSI EP05-A3 single-site precision design (commonly 20 days × 2 runs/day × 2 replicates/run; variants allowed when justified) with variance component estimation for repeatability and within-laboratory precision; report SD/CV by level and across factors such as day/run/operator as applicable.	[98,99]
Accuracy (Trueness)	The closeness of agreement between the average value obtained from a large series of test results and an accepted reference value.	Ensures the result reflects the patient’s true physiological state.	CLSI EP09 method-comparison using split patient samples spanning the shared measuring interval, with visual plots plus regression/bias estimation; EP09-A3 recommends at least 100 patient samples over multiple days for validation/claim verification, with acceptance criteria defined a priori.	[100,101]
LOD	The lowest amount of analyte in a sample which can be detected but not necessarily quantitated as an exact value.	Crucial for early disease diagnosis or detecting low-level biomarkers.	CLSI EP17-A2 detection capability evaluation: estimate LOB from blank results, then estimate LOD from low-level samples using the EP17 parametric framework (LOB = MB + cp·SDB; LOD = LOB + cp·SDL, with cp reflecting the 95th percentile multiplier corrected for finite degrees of freedom); establish LOQ against a predefined accuracy goal (often via precision-profile or CV-based criteria) and report the decision rule.	[102]
Linearity/Measuring Interval	The range of analyte concentrations over which the method gives test results proportional to the concentration of the analyte.	Defines the clinically reportable range of the device.	CLSI EP06 linearity evaluation across the intended measuring interval using multiple concentration levels (historically five to nine levels with multiple measurements per level) and an allowable nonlinearity criterion; report deviation from linear fit by level and define the reportable range supported by the criterion.	[103]
Analytical Specificity (Interference)	The ability of a method to measure only the analyte of interest in the presence of other substances in the sample matrix.	Prevents false positive or negative results from common interferents (e.g., acetaminophen, ascorbic acid, hematocrit effect).	CLSI EP07 interference testing with defined interferent panels and medically relevant challenge concentrations: screen and quantify interferent effects, define a medically significant bias threshold, and verify claims in relevant matrices; report interferent identity, concentration, direction/magnitude of bias, and the decision threshold used.	[104,105]
Stability (Shelf-life and In-use)	The ability of the biosensor to maintain its performance characteristics over a stated period under specified storage and use conditions.	Ensures reliability of the device until its expiration date and during patient use.	CLSI EP25 stability-claim evaluation for IVD reagents/products: real-time (and, when appropriate, accelerated) studies with predefined acceptance criteria tied to the analytical claims (e.g., drift in bias/precision, control recovery), supporting both shelf-life and in-use stability statements.	[106,107]

## Data Availability

No new data were created or analyzed in this study. Data sharing is not applicable to this article.

## Abbreviations

ADC	analog-to-digital converter
Ag/AgCl	silver/silver chloride
AOI	automated optical inspection
APC	article processing charge
ASIC	application-specific integrated circuit
BEOL	back-end-of-line
BLE	Bluetooth Low Energy
CAPA	corrective and preventive action
CD-SEM	critical dimension scanning electron microscopy
CE	Conformité Européenne (CE marking)
CGM	continuous glucose monitoring
CLSI	clinical and laboratory standards institute
CNT(s)	carbon nanotube(s)
CV	cyclic voltammetry
EIS	electrochemical impedance spectroscopy
EOL	end-of-life
EU	European Union
FDA	U.S. Food and Drug Administration
GMP	good manufacturing practice
IQC	incoming quality control
ISO	international organization for standardization
IVD	in vitro diagnostic
IVDD	in vitro diagnostic directive
IVDR	in vitro diagnostic regulation (Regulation (EU) 2017/746)
JIC	just in case (inventory strategy)
JIT	just in time (inventory strategy)
LOD	limit of detection
LOQ	limit of quantification
LOB	limit of blank
MARD	mean absolute relative difference
NMPA	National Medical Products Administration (China)
PC	polycarbonate
PCCP	predetermined change control plan
PER	performance evaluation report
PET	polyethylene terephthalate
PMA	premarket approval
PMPF	post-market performance follow-up
POC	point of care
QMS	quality management system
QSR	quality system regulation
RoHS	restriction of hazardous substances
RSD	relative standard deviation
R^2^	coefficient of determination
R2R	roll-to-roll
SD	standard deviation
SMBG	self-monitoring of blood glucose
SPE	screen-printed electrode
TPP	target product profile
TRL	technology readiness level
UV	ultraviolet
510(k)	510(k) Premarket Notification (U.S. FDA pathway)
